# Research note: Effects of dietary bile acids supplementation on hepatic lipid accumulation and fatty acid profile of hens at late laying cycle

**DOI:** 10.1016/j.psj.2025.106026

**Published:** 2025-10-27

**Authors:** Qingsong Fan, Yilin Ge, Yuemeng Fu, Yufei Gao, Guohui Zhou, Zhenhui Liu, Xuejun Yuan, Weiren Yang, Ning Jiao, Yao Ding, Yang Li

**Affiliations:** a Key Laboratory of Efficient Utilization of Non-grain Feed Resources (Co-construction by Ministry and Province), Ministry of Agriculture and Rural Affairs, Shandong Provincial Key Laboratory of Animal Nutrition and Efficient Feeding, Department of Animal Science and Technology, Shandong Agricultural University, Tai’an 271017, China; b SDU-ANU Joint Science College, Shandong University, Wenhuaxilu, Weihai, China; c College of Life Sciences, Shandong Agricultural University, Daizong Street 61, Tai’an 271018, China; d Shandong Zhongjing Biotechnology Co., Ltd., Tai’an 271411, China

**Keywords:** Additive, Bile acid, Fatty acid, Hens, Liver

## Abstract

The present study was conducted to investigate the effects of dietary bile acids (BAs) addition on hepatic lipid accumulation and fatty acid profile of late-phase laying hens. A total of 1080 healthy 76-week-old Hy-line Brown hens in the late-laying phase were randomly assigned to 4 treatments, and fed a basal diet added with 0 (control), 200 (BA200), 300 (BA300), and 500 (BA500) mg/kg BAs, respectively. The results showed that BAs supplementation significantly decreased steatotic foci and lipid droplets in hepatocytes, minimized vacuoles, and increased normal hepatocytes. Besides, BAs addition reduced the hepatic content of C20:0, C22:0, and C24:0, and up-regulated the mRNA expression levels of *HSL* and *FABP_4_* in the livers of laying hens (linear and quadratic, *P* < 0.05). Moreover, in comparison with the control group, BA200 group significantly elevated C17:0 and C20:2 concentrations (*P* < 0.05), and 300 mg/kg had significantly lower triglyceride content and mRNA expression levels of *FAS* and *SCD* in the liver of laying hens (*P* < 0.05). In conclusion, dietary BAs supplementation decreased liver lipid accumulation and changed fatty acid profile, benefiting the liver health for late-phase laying hens.

## Introduction

The liver serves as the primary organ for lipid metabolism, particularly in chickens, where it is responsible for over 90 % of the de novo synthesis of fatty acids (**FAs**) (Gu et al., 2021). During periods of egg-laying, substantial quantities of triacylglycerols, cholesteryl esters, and free fatty acids (**FFAs**) are synthesized and incorporated into egg-yolk precursors, such as vitellogenin and very low-density lipoprotein, within the liver (Zhao et al., 2024). These compounds are subsequently secreted into the bloodstream, transported to the ovary, and taken up via receptor-mediated endocytosis by the developing oocytes. However, in laying hens during the late production phase, the metabolic efficiency declines, leading to impaired fat mobilization and increased susceptibility to hepatic lipid accumulation (Li et al., 2015; Gu et al., 2021). Excessive fat deposition in the liver can result in fatty liver hemorrhagic syndrome (**FLHS**), a common metabolic disorder that reduces egg production, compromises egg quality, and increases mortality. Additionally, impaired liver function disrupts yolk precursor synthesis, further affecting reproductive performance (Li et al., 2015). Understanding and regulating hepatic lipid metabolism in late-laying hens is essential for maintaining liver health, prolonging peak production, and ensuring sustainable poultry farming.

Bile acids (**BAs**), which are hydroxylated steroids produced in the liver from cholesterol, are commonly utilized as dietary fat emulsifiers in animal feed due to their essential role in lipid metabolism. Previous studies have demonstrated that BA disorders are involved in the development of fatty liver (Zhao et al., 2024), and changing bile acid profile may help enhance fat utilization, mitigate liver steatosis, and improve overall flock productivity in late laying hens (Sun et al., 2023; Xing et al., 2025). While BA supplementation in late-laying hen diets has been recently investigated, inconsistent results persist due to the composition of BAs, age of laying hens, and duration of feeding (Wang et al., 2024; Xing et al., 2025). Therefore, the optimal dosage of BAs in the diets of late laying hens needs to be further studied. Moreover, there is little information available in the scientific literature on the evaluation of impacts of dietary BAs addition on the liver FFA profile which serves as early indicators of hepatic lipid accumulation, predicting the risk of FLHS.

Therefore, the current study aimed to explore the impacts of dietary BAs addition on the lipid accumulation and FFA profile of liver in the hens at late laying cycle, providing useful insights for the application of BAs in poultry production.

## Materials and methods

### Ethical statement

The current study was carried out at the Family Farm of Kongjia Village of Tai’an city (Shandong, China). All the animals and procedures were approved by the Care and Use Committee of Shandong Agricultural University (SDAUA-2023-817).

### BA product

The BA product used in this study was derived from porcine sources, and provided by the Shandong Zhongjing Biotechnology Co., Ltd. Its main active components accounted for 21.53 %, specifically comprising 3.05 % chenodeoxycholic acid (CDCA), 6.59 % phocaecholic acid, 0.24 % hyocholic acid, 0.49 % hyodeoxycholic acid (HDCA), and 11.16 % cholic acid. The product was formulated using wheat flour as premix carrier.

### Animals and treatments

A total of 1080 healthy 76-week-old Hy-line Brown hens in the late-laying phase were randomly assigned to 4 treatments with 6 replicates per treatment and 45 hens per replicate. The layer hens were fed a basal diet added with BAs at dosages of 0 (control), 200 (BA200), 300 (BA300), and 500 (BA500) mg/kg, which corresponded to BAs inclusion rates of 0, 43.06, 64.59, and 107.65 mg/kg of diet, respectively. The trial lasted 35 days. All the hens were housed in three-tiered, wire-floored battery cages (220 × 45 × 45 cm) with *ad libitum* access to feed and water.

### Sampling

After the trial, 2 healthy laying hens close to the average body weight were selected from each replicate, totaling 12 hens per treatment. After the hens were euthanized via cervical dislocation, approximately 5 g samples were collected from the left lobe of the liver after rinsing with physiological saline and blot-drying with filter paper. One part of the samples was minced and aliquoted into 2 mL cryovials, and flash-frozen in liquid nitrogen followed by storage at −80°C; another part was fixed in 4 % paraformaldehyde solution.

### Determination of hepatic lipid and malondialdehyde (MDA) levels

Liver tissue samples were homogenized and centrifuged at 13,500 g for 10 min at 4°C at a 1:9 (w/v) ratio in physiological saline, and the supernatant was collected for subsequent analysis. Hepatic triglyceride (**TC**; CAS#A110-1-1), total cholesterol (**TG**; CAS#A111-1-1), and MDA (CAS#A003-1-2) levels were measured using the commercial assay kits (Nanjing Jiancheng Bioengineering Institute, Nanjing, China) according to the manufacturer's protocols.

### Determination of hepatic FFA profile

The FFA profile of liver samples was analyzed using an ultra-high performance liquid chromatography-tandem mass spectrometry (UPLC-MS/MS) platform provided by Beijing Hexin Technology Co., Ltd. In summary, 50 mg thawed liver samples were homogenized in 1.0 mL of isopropanol:acetonitrile (1:1, v/v). The mixture was vortexed vigorously and extracted for 1 h with continuous shaking, and centrifuged at 13,500 g for 10 min at 4°C. Afterwards, a 95 μL aliquot of the supernatant was mixed with 5 μL of internal standard solution (nonadecanoic acid, FFA 19:0, 10 μg/mL in methanol). Finally, the mixture was transferred to an autosampler vial (fitted with a 250 μL low-volume insert) and subjected to LC-MS analysis using Waters ACQUITY UPLC I-CLASS System (Waters, Switzerland) with Waters Xevo TQ-S micro mass spectrometry. The chromatographic separation was achieved using a Waters UPLC BEH C8 mass column (2.1 × 100 mm, 1.7 µm particle size) at a flow rate of 0.26 mL/min. The quantification of individual FAs was performed by measuring peak areas and the results were ex-pressed as a percentage of the total FAs.

### Examination of hepatic histopathology

After fixation in 4 % paraformaldehyde for 24 h, the liver samples were dehydrated through a graded ethanol series, and embedded in paraffin using standard histological protocols. Subsequently, 5-µm-thick sections were prepared and stained with hematoxylin and eosin (H&E). Histological observations were performed using an Olympus BX51 digital microscope (Tokyo, Japan).

### Quantitative real-time PCR analysis

The hepatic mRNA expression levels of lipid metabolism-related genes, including hormone-sensitive lipase (***HSL***), stearoyl-CoA desaturase (***SCD***), lipoprotein lipase (***LPL***), fatty acid synthase (***FAS***), fatty acid-binding protein 4 (***FABP_4_***) and adipocyte orientation and differentiation factor 1 (***ADD_1_***), were determined by real-time fluorescent quantitative polymerase chain reaction (qPCR) according to a previous method (Sun et al*.*, 2023).

### Statistical analysis

Data were analyzed in SAS9.4 software using one-way ANOVA, combined with Tukey's method for multiple comparisons. Polynomial contrasts were used to assess the linear and quadratic effects of dietary BAs addition. Results are presented as mean ± SE in figures and as means with the SEM in tables, with *P* < 0.05 indicating a significant difference.

## Results and discussion

**Fig. 1:**
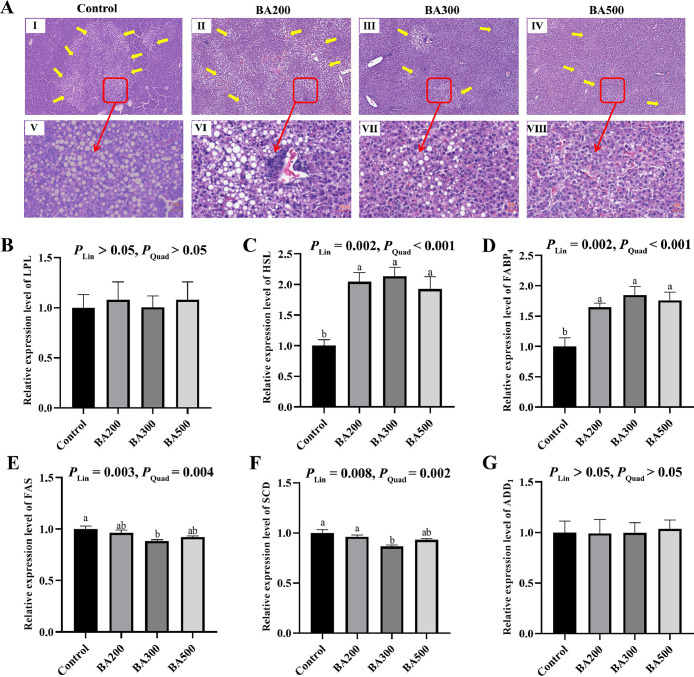
Effects of bile acids supplementation on liver histopathology and mRNA expression of lipid metabolism-related genes in the hens at late laying cycle. (A) Hematoxylin and eosin photomicrographs obtained at 40 × (Ⅰ-Ⅳ) and 400 × (Ⅴ-Ⅷ) magnification; (B) *HSL*, hormone-sensitive triglyceride lipase; (C) *SCD*, stearoyl-CoA desaturase-1; (D) *ADD1*, adipocyte determination and differentiation factor-1; (E) *LPL*, lipoprteinlipase; (F) *FAS*, fatty acid synthetase; (G) *FABP4*, fatty acid-binding protein 4. Control, BA200, BA300, and BA500 represent the basal diet added with 0, 200, 300, and 500 mg/kg bile acids, respectively. ^a,b^ Means with different lowercase letters differ significantly among the four treatments (*P* < 0.05). Lin and Quad showed linear and quadratic effects of different dietary BAs levels. *n* = 6.

During the egg-laying period, the hepatic system of hens frequently functions under substantial metabolic stress, and increasing age can adversely affect liver metabolism and function of laying hens (Gu et al., 2021). Hepatic TG content serves as a critical biomarker for assessing lipid metabolism. In this study, compared with the control group, the liver TG content (Table 1) was significantly reduced in the BA300 group (*P* < 0.05), and showed linear and quadratic decrease (*P* < 0.05) with the BAs addition increasing. The addition of 100 and 200 mg/kg BAs to the diet showed no significant effect on hepatic TC and TG content in the current study (*P* > 0.05), which was consistent with the results in Wang et al*.* (2024). However, Xing et al. (2025) indicated that dietary inclusion of BAs at concentrations of 120 mg/kg (62-69 weeks) and 200 mg/kg (70-75 weeks), comprising 68.31 % HDCA, 7.73 % HCA, and 18.96 % CDCA, resulted in a reduction of TC and TG concentrations in the liver of late laying hens. Similarly, Sun et al. (2023) showed the TG and TC content of liver was reduced by addition of 100 and 200 mg/kg BAs, consisting of 77.2 % HCA and HDCA and 19.9 % CDCA, in laying hens aged 50 to 58 weeks. The discrepancy between the results of these studies might stem from the differences in the types and the absolute amounts of BAs added to the diets and the duration of feeding. The mechanism by which BAs regulate TG metabolism in late laying hens requires further investigation. Besides, the liver morphological results demonstrated that compared with the control group, BAs supplementation led to a significant reduction in steatotic foci, smaller and fewer vacuoles, decreased lipid droplets in hepatocytes, and increased number of normal hepatocytes (Fig. 1A). Previous studies also indicated that dietary BA reduced the diameter and area of vacuoles and lipid droplet content in livers of laying hens (Sun et al., 2023; Wang et al., 2024). Moreover, BA300 and BA500 groups showed distinctly visible liver cord structures and sinusoids, clear cytoplasm and nuclei separation, and uniform, well-organized hepatocytes with no significant inflammation detected. Therefore, these results demonstrated that 300 mg/kg BAs supplementation could effectively reduce liver TG content and hepatic steatosis, and partially restore normal liver architecture and function.

**Table 1:** Effects of bile acids (BAs) supplementation on the fatty acid (FA) profile in the liver of hens at late laying cycle.

Items 2	Treatments 1				SEM	*P* value 3		
	Control	BA200	BA300	BA500		BA	Lin	Quad
TG (mmol/g prot)	0.07^a^	0.05^ab^	0.04^b^	0.05^ab^	0.003	0.016	0.013	0.005
TC (mmol/g prot)	0.22	0.21	0.20	0.20	0.004	0.381	0.077	0.217
MDA (nmol/mg prot)	7.57^a^	7.35^ab^	6.67^c^	6.94^bc^	0.09	<0.001	<0.001	<0.001
FFA (μg/g)								
C11:0	1.82	2.16	1.96	2.02	0.11	0.781	0.647	0.749
C12:0	19.31	20.50	24.31	23.55	1.54	0.642	0.270	0.526
C14:0	134.42	109.56	144.89	134.92	10.48	0.699	0.808	0.917
C15:0	4.53	4.89	5.17	5.27	0.34	0.883	0.423	0.719
C16:0	483.17	529.93	451.34	450.25	20.14	0.485	0.414	0.611
C17:0	10.32^b^	15.97^a^	10.58^b^	11.68^b^	0.68	0.004	0.888	0.256
C18:0	311.79	372.67	317.41	342.17	11.08	0.196	0.557	0.619
C20:0	2.63^a^	2.16^b^	1.93^bc^	1.76^c^	0.08	<0.001	<0.001	<0.001
C21:0	0.88	0.68	0.70	0.63	0.04	0.060	0.014	0.031
C22:0	0.35^a^	0.22^b^	0.23^b^	0.19^b^	0.02	0.013	0.004	0.006
C23:0	0.04	0.03	0.03	0.02	0.001	0.082	0.010	0.038
C24:0	0.11^a^	0.08^b^	0.08^b^	0.06^b^	0.01	0.045	0.004	0.016
SFA	969.36	1058.85	958.61	972.54	36.38	0.773	0.876	0.873
C14:1	67.51	51.84	69.29	61.85	4.27	0.493	0.864	0.886
C16:1	535.31	468.50	610.19	576.35	41.15	0.683	0.628	0.864
C17:1	23.80	24.41	29.17	26.66	1.63	0.668	0.429	0.661
C18:1	680.94	866.79	743.37	734.70	30.69	0.178	0.751	0.281
C20:1	13.97	18.26	17.93	16.32	0.72	0.128	0.281	0.059
C22:1	2.14	2.17	2.08	2.15	0.19	0.999	0.980	0.998
C24:1	0.39	0.34	0.40	0.38	0.03	0.888	0.984	0.973
MUFA	1334.07	1432.31	1472.44	1418.40	60.86	0.892	0.610	0.734
C18:2	1025.61	1133.17	1034.93	1081.24	63.36	0.938	0.849	0.957
C18:3	170.42	137.56	150.41	141.91	11.79	0.790	0.458	0.674
C18:4	0.37	0.33	0.31	0.38	0.03	0.804	0.980	0.615
C20:2	7.01^b^	10.93^a^	7.06^b^	6.65^b^	0.54	0.006	0.476	0.099
C20:3	55.15	58.53	46.60	42.90	3.08	0.251	0.101	0.226
C20:4	528.62	560.72	544.98	536.05	31.74	0.988	0.964	0.952
C20:5	4.42	5.15	4.70	6.70	0.59	0.559	0.207	0.399
C22:2	0.59	0.84	0.72	0.70	0.04	0.116	0.406	0.128
C22:3	1.53	2.42	2.04	1.81	0.13	0.090	0.608	0.082
C22:4	10.50	14.42	9.74	8.98	0.78	0.053	0.286	0.183
C22:5	57.69	45.10	52.07	44.15	2.82	0.292	0.138	0.313
C22:6	172.07	184.27	186.55	212.18	17.00	0.881	0.420	0.715
PUFA	2034.00	2153.42	2020.11	2083.63	118.75	0.986	0.939	0.986
USFA	3368.07	3585.72	3512.55	3502.03	172.18	0.980	0.816	0.926
Total	4337.43	4644.58	4471.17	4474.57	206.06	0.969	0.868	0.927

1 Control, BA200, BA300, and BA500 represent the basal diet added with 0, 200, 300, and 500 mg/kg bile acids, respectively.

2 TG, triglyceride; TC, total cholesterol; MDA, malondialdehyde; FFA, free fatty acid; SFA, saturated fatty acid; USFA, unsaturated fatty acid; MUFA, monounsaturated fatty acid; PUFA, polyunsaturated fatty acids.

3 Lin and Quad showed linear and quadratic effects of different dietary BAs levels. ^a,b,c^Means with different lowercase letters differ significantly among the four treatments (*P* < 0.05). *n* = 6.

In contrast, compared with the control group, dietary inclusion with BAs significantly decreased the hepatic content of C20:0, C22:0, and C24:0 (linear and quadratic, *P* < 0.001; Table 1). Saturated long-chain FAs (**SLCFAs**) has been demonstrated high hepatotoxicity. Very-long-chain FA elongases (**VLCFAE**) are responsible for extending the carbon chain of FAs (Wang et al., 2023). The decreases in hepatic C20:0, C22:0, and C24:0 content might suggest an attenuation of VLCFAE by dietary BAs addition, which needed to be further studied. FAS is a critical enzyme essential for the de novo biosynthesis of long-chain FAs. However, only BA300 group had a significantly lower mRNA expression level of *FAS* (Fig. 1E) than the control group in this study (*P* < 0.05). Hepatic lipid metabolism is governed by de novo lipogenesis, fatty acid uptake/export, and fat utilization by β-oxidation. We found that dietary BAs addition significantly up-regulated the mRNA expression levels of *HSL* (Fig. 1C) and FABP_4_ (Fig. 1D) in the livers of laying hens relative to the control group (*P* < 0.05). HSL, a critical rate-determining enzyme of lipolysis in adipocytes, hydrolyzes TGs into FFAs and glycerol in response to hormonal signals. The increased HSL expression in the liver could also contribute to the decreased hepatic TG level by BAs addition. FABP_4_ is a FA transporter that coordinates lipid metabolism, and its overexpression results in the lipid accumulation (Xing et al., 2025). The increased FABP_4_ expression observed in this study was likely a compensatory cellular response to BA-induced lipolysis. This upregulation probably facilitated the transport of FAs towards catabolic pathways, thereby promoting their oxidation and ultimately contributing to the reduction in lipid accumulation. Recent study has shown that the interaction between HSL and FABP_4_ could promote lipolysis in adipocytes (Yu et al., 2024). Moreover, BA300 group showed significantly decreased *SCD* (Fig. 1F) mRNA expression compared with the control group and BA200 group in the present study (*P* < 0.05). SCD is a rate-limiting enzyme that catalyzes the conversion of saturated FAs (**SFAs**) into unsaturated FAs (**USFAs**), thereby increasing the degree of unsaturation of FAs and reducing lipid droplet formation. Wang et al. (2024) also found that inclusion of low-dose (91.05 mg/kg) BAs suppressed hepatic genes expressions involved in lipid synthesis, including *FAS* and *SCD* in late-phase laying hens, compared with the control group. Besides, previous study also demonstrated that dietary BAs addition could activate the Farnesoid X receptor, and then increased the peroxisome proliferator-activated receptor expression, thereby promoting β-oxidation of FAs and reducing the lipogenesis in the liver of laying hens (Sun et al., 2023; Zhao et al., 2024). Therefore, dietary BAs supplementation could decrease hepatic concentrations of SLCFAs through promoting the lipolysis and FFA transport and reducing the lipogenesis in late-phase laying hens, with the 300 mg/kg dosages exhibiting superior efficacy. However, the underlying mechanism by which BAs regulate hepatic SLCFAs in late laying hens needs to be further verified.

Interestingly, we found dietary inclusion with 200 mg/kg BAs significantly elevated C17:0 and C20:2 concentrations in comparison with the control group (*P* < 0.05, Table 1). Higher levels of odd chain SFAs, such as C15:0 and C17:0, are inversely associated with risk of metabolic disease. C20:2, an n-6 polyunsaturated fatty acid, modulates inflammatory responses and undergoes conversion to sciadonic acid, a fatty acid with anti-inflammatory properties (Huang et al., 2011). The C17:0 formation in the liver can be affected by the bacterial propionate production. Yang et al. (2022) showed that dietary BAs supplementation altered cecal microbiota composition and affected short-chain FA metabolism in late-phase laying hens. Moreover, inclusion of dietary BA could up-regulate USFA biosynthesis and FA degradation by changing gut microbiota of hens during the late laying period (Yang et al*.*, 2022), which might be a reason for the increased C20:2 content and decreased concentrations of C20:0, C22:0, and C24:0 in the liver in this study. Therefore, dietary 200 mg/kg BAs supplementation might help guard against the metabolic disease through the generation of odd chain FA and C20:2 in the liver of hens at late laying cycle. The underlying mechanism requires further investigation.

Lipid metabolism dysfunction is closely associated with oxidative stress, a key mediator of reduced egg production in laying hens (Gu et al*.*, 2021). MDA is one of the final products of PUFAs peroxidation in the cells, serving as a crucial indicator for assessing cellular oxidative stress levels. We found that liver MDA concentration decreased significantly with increasing BA addition (linear and quadratic, *P* < 0.001), with the BA300 group exhibiting the lowest level. Significantly lower MDA concentrations were also found in both the BA300 and BA500 groups compared to the control group (*P* < 0.05). Similarly, Xing et al*.* (2025) demonstrated that dietary addition with BAs reduced hepatic MDA content in late laying hens. Therefore, 300 and 500 mg/kg BAs addition could reduce hepatic oxidative stress response in laying hens.

Collectively, dietary BAs supplementation benefited to improve liver health of late-phase laying hens through decreasing the lipid accumulation and oxidative stress, which might be attributed to the reduction in hepatic concentrations of SLCFAs through promoting the lipolysis and FFA transport and reducing the lipogenesis. Especially, 300 mg/kg BAs supplementation showed the greatest beneficial effects. These findings suggested that the BA mixture could be a potential liver beneficial promoter for late-phase laying hens.

## Disclosures

We declare that we have no conflict of interest to this work.
